# The *CaAP2/ERF064* Regulates Dual Functions in Pepper: Plant Cell Death and Resistance to *Phytophthora capsici*

**DOI:** 10.3390/genes10070541

**Published:** 2019-07-17

**Authors:** Jing-Hao Jin, Huai-Xia Zhang, Muhammad Ali, Ai-Min Wei, De-Xu Luo, Zhen-Hui Gong

**Affiliations:** 1 College of Horticulture, Northwest A&F University, Yangling 712100, China; 2 Tianjin Vegetable Research Center, Tianjin 300192, China; 3 Xuhuai Region Huaiyin Institute of Agricultural Sciences, Huai’an 223001, China

**Keywords:** pepper, AP2/ERF, *P. capsici*, cell death, *PR* gene

## Abstract

*Phytophthora* blight is one of the most destructive diseases of pepper (*Capsicum annuum* L.) globally. The APETALA2/Ethylene Responsive Factors (*AP2/ERF*) genes play a crucial role in plant response to biotic stresses but, to date, have not been studied in the context of *Phytophthora* resistance in pepper. Here, we documented potential roles for the pepper *CaAP2/ERF064* gene in inducing cell death and conferring resistance to *Phytophthora capsici* (*P. capsici*) infection. Results revealed that the N-terminal, AP2 domain, and C-terminal of CaAP2/ERF064 protein is responsible for triggering cell death in *Nicotiana benthamiana (N. benthamiana)*. Moreover, the transcription of *CaAP2/ERF064* in plant is synergistically regulated by the Methyl-Jasmonate (MeJA) and ethephon (ET) signaling pathway. *CaAP2/ERF064* was found to regulate the expression of *CaBPR1*, which is a pathogenesis-related (*PR*) gene of pepper. Furthermore, the silencing of *CaAP2/ERF064* compromised the pepper plant resistance to *P.* *capsici* by reducing the transcript level of defense-related genes *CaBPR1*, *CaPO2*, and *CaSAR82*, while the ectopic expression of *CaAP2/ERF064* in *N. benthamiana* plant elevated the expression level of *NbPR1b* and enhanced resistance to *P.* *capsici*. These results suggest that *CaAP2/ERF064* could positively regulate the defense response against *P. capsici* by modulating the transcription of *PR* genes in the plant.

## 1. Introduction

Plants are continuously exposed to various pathogens and have evolved complex mechanisms to regulate defense response [1,2]. Members of the APETALA2/Ethylene Responsive Factor (*AP2/ERF*) gene family play an important role in biotic and abiotic defense responses in plants [3,4]. In a previous study in tobacco, *ERF* genes were identified through binding to the GCC-Box cis-element of the pathogenesis-related (*PR*) genes [5]. In *Arabidopsis*, *AtERF96* promotes the expression levels of several defense genes (*PDF1.2*, *PR-3*, and *PR-4*), by directly binding to GCC-Box elements of their promoters, and enhances the plant resistance to necrotrophic pathogens [6]. Furthermore, it was found that the expression of the ERF gene *RAP2.2* was upregulated upon the infection of *Botrytis cinerea* in *Arabidopsis*, while this upregulation was disrupted in *ethylene insensitive 2* (*ein2*) and *ein3* mutants. Moreover, the over-expression of *RAP2.2* in *ein2* and *ein3* mutants restored the *Arabidopsis* plant resistance to *Botrytis cinerea* [7]. It has also been shown that the *Arabidopsis ERF5* gene promotes plant defense responses to multiple pathogens through coordinating chitin pathways [8].

A subset of AP2/ERF factors contains an ERF-associated amphiphilic repression (EAR) domain and have been shown to function as a transcriptional repressors of defense-related genes [9,10]. For example, *AtERF4* was found to negatively regulate plant response to *Fusarium oxysporum* infection in *Arabidopsis* [9], while *StERF3* negatively regulates plant resistance to *Phytophthora infestans* and salt stress [11], whereas the over-expression of *GmERF5* increases the transcription level of *PR* genes and also enhances plant resistance to *Phytophthora sojae* infection [12]. Recently, it was found that *AtERF8* gene positively regulates plant resistance to *Pseudomonas syringae* [13,14].

In previous studies, several ERF genes were identified in the pepper plant. *CaERFLP1* was transcriptionally activated during the infection of pathogens in pepper [15]. The over-expression of *CaPF1* in *Arabidopsis* and tobacco plants increased resistance to *Pseudomonas syringae* [16], whereas the *CaPTI1* gene has been shown to play a crucial role in resistance to *Phytophthora capsici* (*P. capsici*) infection [17]. Recently, the *CaAIEF1* gene was found to regulate the response to drought stress in plants [18].

Oomycetes include a large number of pathogens. *P. capsici* causes root and fruit rot on pepper and other vegetables (tomato, cucurbits, and lima beans) plant. *P. capsici* is a highly destructive pathogen and is considered to be one of the most significant oomycete pathogens limiting production of these vegetables [19,20]. Members of the AP2/ERF gene family have been reported to play a key role in plant response to different pathogens. However, little is known about the exact role of *CaAP2/ERF* genes in pepper plant response to *P. capsici*.

In the previous study, the AP2/ERF family genes were identified from the latest pepper genome database in response to *P. capsici* infection and various phytohormones treatments. Moreover, one of these genes, *CaAP2/ERF064*, was found to respond to the infection of *P. capsici*, and CaAP2/ERF064 protein was located in the nucleus [21]. In this study, the expression pattern of *CaAP2/ERF064* in response to various combinations of phytohormones was investigated with qRT-PCR. Moreover, its function in plant defense response to *P. capsici* was elucidated by the virus-induced gene silencing (VIGS) technique and ectopic transformation in *Nicotiana benthamiana (N. benthamiana).* Additionally, the transcription regulation relationship between *CaAP2/ERF064* and the *CaBPR1* gene was also identified with a yeast one-hybrid assay. The cell’s death, which induced by the over-expression of *CaAP2/ERF064* and RNA silencing suppressor *P19*, was investigated with a transient expression assay in *N. benthamiana*.

## 2. Materials and Methods

### 2.1. Plant Materials and Phytohormones Treatments

The *Phytophthora capsici*-resistant pepper (*Capsicum annuum* L.) lines Y5 was obtained from Vegetable Plant Biotechnology and Germplasm Innovation lab, Northwest A&F University-China. Pepper seedlings were grown under 16 h light and 8 h dark photoperiods, with 25 and 18 °C day and night temperatures, respectively, and 65% relative humidity. *Nicotiana benthamiana* plants were grown under 16 h light and 8 h dark photoperiods, with 22 and 20 °C day and night temperatures, respectively, and 65% relative humidity.

For phytohormones treatments, the Y5 plants, at 6–8 leaves stage, were sprayed with 1mM salicylic acid (SA), 100 μM Methyl-Jasmonate (MeJA) or 100 μM ethephon (ET). Control plants were sprayed with mock solution. Leaf samples were collected at 0, 3, 6, and 12 h post-treatment (hpt).

### 2.2. Pathogen Preparation and Inoculation

*P. capsici* strain HX-9 was obtained from our laboratory, and zoospores of *P. capsici* were prepared as previously described [21,22]. Briefly, *P. capsici* on potato dextrose agar (PDA)medium was divided into pieces and covered with liquid carrot broth (200 g/L) in a Petri dish for three days at 28 °C. Then, the cultures were washed with sterile water and covered with Petri broth for five days in the dark. To release zoospores, cultures with sporangia were covered with cold sterile water and placed at 4 °C for 30 min. Finally, the concentration of zoospores was adjusted to 1 × 10^6^ zoospores/mL with sterile water.

For the detached leaf assay, 10 μL zoospores suspension was applied on the dorsal side of each leaf, and the leaves were then incubated on 22–25 °C [23], while for the inoculation of tobacco plants, 5 mL zoospores suspension was applied to each plant by a foliar and root drench, and the plants were then incubated at 25 °C [24]. The leave samples were collected at 0, 3, and 7 days post-inoculation (dpi). The disease index of tobacco plant was determined at 0, 7, and 14 dpi, as described in our previous paper [24], while symptoms on tobacco plants are characterized into different levels (L_0_–L_6_) which are: Level_0_: no symptoms; Level_1_: stem shrink < 1 cm (from the base); Level_2_: >1–2 cm and leaves wilting; Level_3_: > 2–3 cm and leaves become yellow; Level_4_: > 3–4 cm and leaves become yellow; Level_5_: > 4–5 cm and the leaves become yellow and withered; and Level_6_: > 5 cm or whole plant died.

### 2.3. Transient Expression Assay in Tobacco

To construct the transient expression vector, the coding sequence (CDS)and deletion fragments of *CaAP2/ERF064* gene and its homologs (*StERF1B-l*, *SlTSRF1*, and *NbERF1B-l*) (excluding stop-codon) were cloned into a pCAMBIA3301-green fluorescent protein (GFP) vector, while the *PcINF1* and *NbCD1* were used as positive controls (Table S1). The recombinant constructs were transformed into *Agrobacterium tumefaciens* (GV3101) with the freeze-thaw method.

The transient expression assay was performed as described in our previous study [21]. Briefly, Agrobacterium strains were grown in Luria-Bertani (LB) liquid medium at 28 °C for 12–16 h, then collected by centrifugation and suspended with the infiltration medium (10 mM MgCl_2_, 10 mM MES, pH 5.5, 200 µM acetosyringone) to an OD_600_ of 0.8. The suspension cells were mixed in a 1:1 ratio with *P19* (RNA-silencing suppressor), and then the mixture was injected in *N. benthamiana* leaves through a needleless syringe. Cell death was monitored at 4–7 days post infiltration. For the transient expression of CaAP2/ERF064 and derived deletion mutants in Pro_CaBPR1_:CaBPR1 transgenic tobacco plants, infiltration was done identically, and samples were collected 3 days after infiltration.

While for the subcellular localization of different homologs and deletion mutants of *CaAP2/ERF064* gene, the GFP signal in infiltrated tobacco leaves was detected with OLYMPUS BX63 (OLYMPUS, Tokyo, Japan) fluorescence microscope at 2–3 days post-infiltration.

### 2.4. Transcriptional Activation and Yeast One-Hybrid Assay

To evaluate transcriptional activation, the *CaAP2/ERF064* coding sequence and derived deletion fragments were cloned into the pGBKT7 vector. The recombination constructs were transformed into yeast strain (Y 2-H gold) according to the manufacturer’s protocol (Clontech, CA, U.S.). The resulting strains were grown on SD/-Trp, SD/-Trp-His-Ade, and SD/-Trp-His-Ade + X-α-Gal at 30 °C for 2–3 days.

For yeast one-hybrid assay, the CDS of *CaAP2/ERF064* and the promoter fragment of *CaBPR1* were cloned into the pGADT7 and pHIS2 vector, respectively. Then, the resulting strains (Y187), which contained these constructs, were grown on SD/-Leu-Trp and SD/-Leu-Trp-His medium contained 90 mM 3-AT (the minimal inhibitory concentration of the promoter activation of *CaBPR1*) at 30 °C for three days.

### 2.5. Virus-Induced Gene Silencing in Pepper

For VIGS assays, a 267-bp specific fragment of *CaAP2/ERF064* was cloned into pTRV2 to generate the TRV2:CaAP2/ERF064 vector. VIGS was performed as in our previous study [25]. Briefly, Agrobacterium strains (TRV2:CaAP2/ERF064, TRV2:GFP, and TRV2:CaPDS), as well as pTRV1, were grown in induction medium at 28 °C for 12–18 h; then cells were collected by centrifugation and suspended with the infiltration medium (10 mM MgCl_2_, 10 mM MES, pH 5.5, 200 µM acetosyringone). The suspension was mixed in a 1:1 ratio with suspension of pTRV1 and used for infiltration of two-weeks-old pepper seedlings. Finally, the pepper plants were incubated in the chamber at 22 °C.

### 2.6. Tobacco Transformation

The CDS of *CaAP2/ERF064* was cloned into pCAMBIA2300-RC to generate the pCaMV35S:CaAP2/ERF064 vector., while the promoter (2016-bp) and CDS fragment of *CaBPR1* gene (excluding stop-codon) was cloned into the pBI121 vector. Both of these constructs were transformed into *Agrobacterium tumefaciens* (GV3101) and then used for the tobacco transformation. The pCaMV35S:CaAP2/ERF064 and Pro_CaBPR1_:CaBPR1 transgenic tobacco plants were generated with the Agrobacterium-mediated leaf disc transformation assay [26]. All the transgenic tobacco plants were confirmed with qRT-PCR and T_2_ generation plants were used for further analysis.

### 2.7. Histochemical Staining

3,3-diaminobenzidine (DAB) and trypan-blue staining were conducted as described by Choi, et al. [27]. For DAB staining, the tobacco leaves were incubated in DAB solution (1 mg mL^−1^, pH 3.8) for 8–10 h. Then, the leaves were cleared with 75% ethanol, while for trypan-blue staining, pepper and tobacco leaves were boiled in trypan-blue solution (10 mL lactic acid, 10 mL glycerol, 10 g phenol, and 10 mg trypan blue, dissolved in 10 mL distilled water) for 2–5 min. Then, leaves were de-stained with chloral hydrate (2.5 g mL^−1^).

### 2.8. qRT-PCR Analysis

For gene expression analysis by qRT-PCR, total RNA was isolated with the Trizol method as previously described [24], and cDNA was synthesized with reverse transcript kit according to the manufacturer’s instructions (Vazyme, China). The qRT-PCR assay was performed as previously described [21,24]. The β-glucuronidase (GUS) gene primers were used to detect the expression level of *CaBPR1* in Pro_CaBPR1_:CaBPR1 transgenic tobacco plants. *CaUBI3* and *NbEF1α* were used as reference genes in pepper or tobacco, respectively. The relative expression levels of each gene were calculated by the 2^−∆∆Ct^ method [28]. All the primers used for qRT-PCR are listed in Table S1.

### 2.9. Statistical Analysis

Data Processing System software was used for statistical analysis with least significant difference (LSD) at *p* < 0.05 levels. All experiments were performed and analyzed independently with at least three biological replicates.

## 3. Results

### 3.1. Expression Analysis of CaAP2/ERF064 in Response to Combinations of Phytohormones

**Figure 1:**
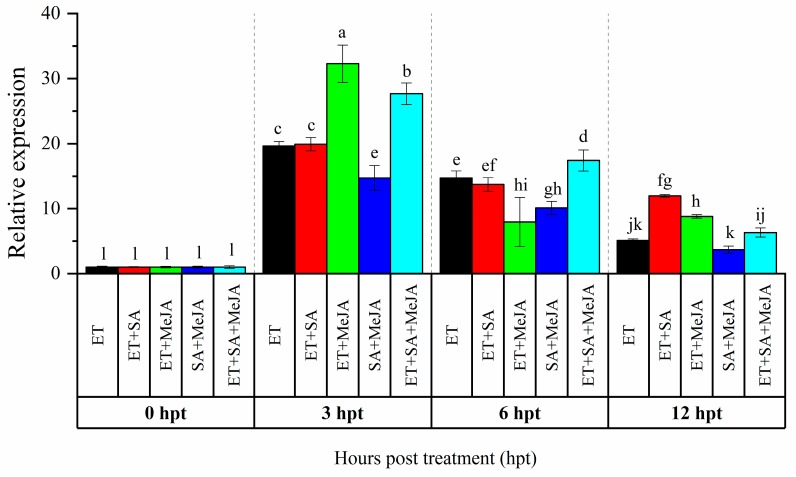
Expression analysis of *CaAP2/ERF064* in response to various combinations of phytohormones. Values are means ± SD. Small letters indicate a significant difference (least significant difference (LSD), *p* < 0.05).

Our previous study showed that *CaAP2/ERF064* gene exhibited a high response to the treatment of SA, MeJA, and ET [21]. To investigate potential for cross-talk signaling in the regulation of *CaAP2/ERF064*, we treated pepper plants with combinations of these phytohormones. As shown in Figure 1, with ET treatment, the highest (19.65-fold) and lowest (5.08-fold) expression level of *CaAP2/ERF064* were detected at 3 and 12 h post-treatment (hpt), respectively, while the expression level of *CaAP2/ERF064* in treatment with ET + SA, ET + MeJA, SA + MeJA, and ET + SA + JA peaked at 3 hpt, which is 19.91, 32.29, 14.73, and 27.67-fold, respectively. The expression level of *CaAP2/ERF064* in treatment of ET + MeJA (32.29-fold) and ET + SA + MeJA (27.67-fold) was higher than that in treatment of ET (19.65-fold) and MeJA (2.64-fold) [21] at 3 hpt, while in the SA + MeJA treatment, the response was lower than that in the ET treatment. These results indicated that MeJA augments the effect of ET on the transcription of *CaAP2/ERF064* gene in the pepper.

### 3.2. Transient Over-Expression of CaAP2/ERF064 Induces Cell Death in N. benthamiana

**Figure 2:**
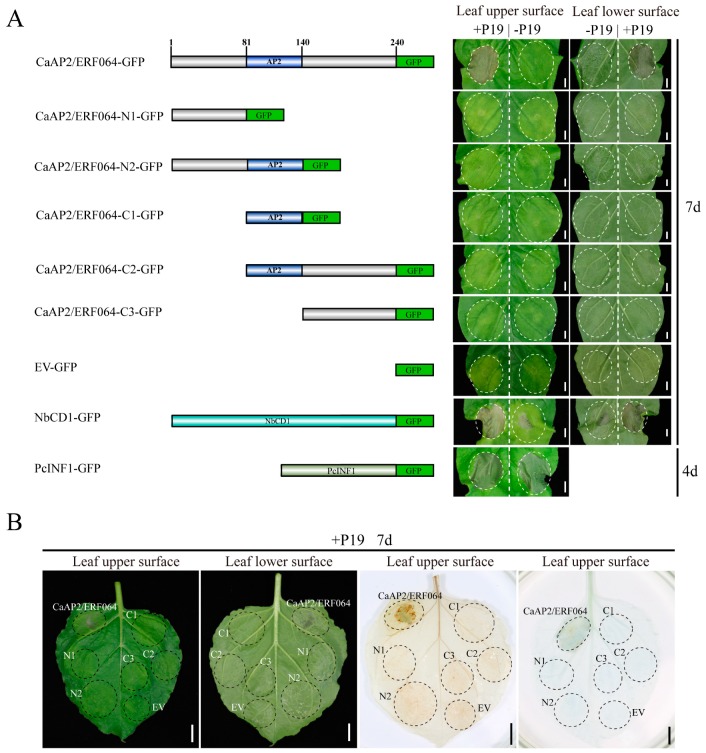
Transient over-expression of *CaAP2/ERF064* triggers cell death in *Nicotiana benthamiana (N. benthamiana)*. (**A**) *CaAP2/ERF064* and its deletion mutants in *N. benthamiana*. Scale bar represents 0.5 cm. (**B**) 3,3-diaminobenzidine (DAB) and trypan-blue staining of tobacco leaves. Scale bar represents 1 cm.

We found that the tobacco leaves co-infiltrated with *CaAP2/ERF064* and *P19* (suppressor of RNA silencing) [29,30] exhibited cell death phenotypes in the infiltrated area (Figure 2). The cell death area was confirmed by using trypan-blue staining (Figure 2B). Furthermore, to identify the domains of *CaAP2/ERF064* responsible for the induction of cell death, different deletion mutants of *CaAP2/ERF064* were tested for their potency in cell death upon transient over-expression by agro-infiltration. Results showed that its deletion mutant N2 induced the collapse of the epidermis but not cell death in tobacco leaves (Figure 2). The other deletion mutants and empty vector (EV) control did not induce any obvious symptoms in the infiltration area. In the absence of *P19*, both *CaAP2/ERF064* and its N2-mutant could induce the collapse of the epidermis in leaves. The positive control, *NbCD1*, also caused cell death when over-expressed in tobacco. However, the death symptom caused by *NbCD1* was weaker when *P19* was absent. In contrast, the elicitor of *P. capsici*, *PcINF1*, induced strong hypersensitive cell death in the leaves at four days post-infiltration.

**Figure 3:**
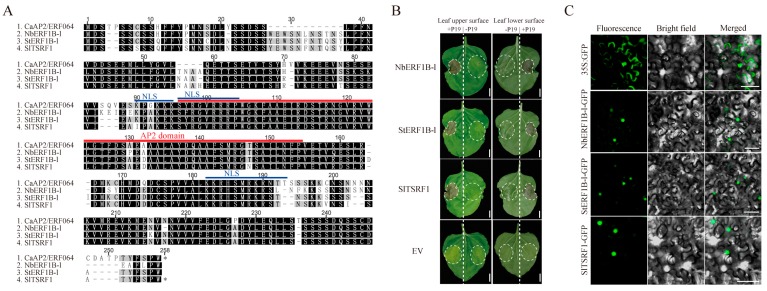
Induction of cell death by CaAP2/ERF064 homologues in *N. benthamiana*. (**A**) Sequence alignment of CaAP2/ERF064 and its homology proteins. (**B**) Transient over-expression of StERF1B-l, NbERF1B-l, and SlTSRF1 in *N. benthamiana*. EV was short for empty vector. Scale bar represents 1 cm. The photograph was taken at seven days post-infiltration. (**C**) Localization of StERF1B-l, NbERF1B-l, SlTSRF1 proteins in *N. benthamiana* epidermal cells. The scale bar represents 50 μm.

Furthermore, the protein localization of different mutants of CaAP2/ERF064 was detected with a fluorescence microscope. Results showed that CaAP2/ERF064 and its N2 mutant proteins have a strong fluorescence signal in the nucleus (Figure S1) [21], whereas the deletion mutant C1 protein was located in the cytoplasm, and the N1, C2, and C3 mutant proteins were detected in both the cytoplasm and nucleus. The homologs (*StERF1B-l*, *SlTSRF1*, and *NbERF1B-l*) of *CaAP2/ERF064* also caused cell death when co-expressed with *P19* in tobacco leaves (Figure 3A,B), and all showed localization in the nucleus (Figure 3C). Moreover, other ERF genes (*CaAP2/ERF049* and *CaAP2/ERF109*) did not induce the cell death when co-overexpressed with *P19* in tobacco plants (Figure S2). These results suggested that transient over-expression of *CaAP2/ERF064* induces cell death, and its N-terminal, AP2 domain, and C-terminal are necessary to trigger cell death in *N. benthamiana*.

### 3.3. CaAP2/ERF064 Regulates the Expression of CaBPR1

**Figure 4:**
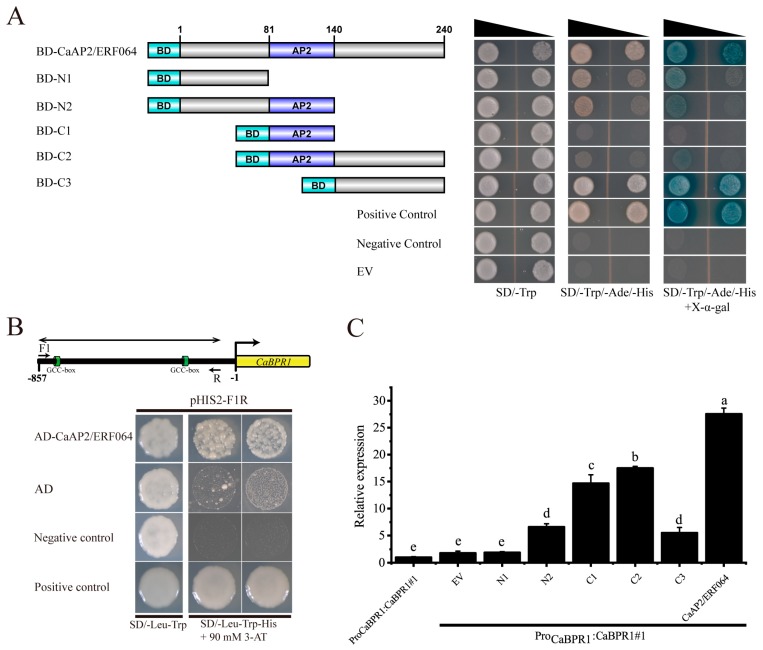
*CaAP2/ERF064* promotes the transcription of *CaBPR1*. (**A**) Transcriptional activation of CaAP2/ERF064 in yeast. (**B**) Interaction of CaAP2/ERF064 and the promoter fragments (F1R) of *CaBPR1* in yeast. (**C**) Expression analysis of *CaBPR1* in Pro_CaBPR1_:CaBPR1 transgenic tobacco plants after infiltration of *CaAP2/ERF064* and its deletion mutants. Values are means ± SD. Small letters indicate a significant difference (LSD, *p* < 0.05).

To determine if CaAP2/ERF064 protein might function as a transcription factor in pepper, its transcriptional activity was evaluated in yeast. As shown in Figure 4A, the yeast stain containing BD-CaAP2/ERF064 protein showed activation of reporter gene LacZ and grew well in the medium of SD/-Trp-His-Ade and SD/-Trp-His-Ade + X-α-Gal. The deletion mutant C3 of CaAP2/ERF064 also exhibited strong transcription activity, while the deletion mutants N1, N2, and C2 showed weak activity, and the C2 mutant showed no activity in yeast. These results indicated that the CaAP2/ERF064 protein can act as a transcriptional activator.

Furthermore, ERF transcription factors can bind to the GCC-box element of target genes, and the *CaBPR1* (PR1 gene) contains two GCC-box elements in the promoter region [31]. Then, the yeast one-hybrid experiment was performed to detect whether CaAP2/ERF064 protein could bind to the promoter fragment (F1R) of the *CaBPR1* gene. The results showed that the yeast strain containing AD-CaAP2/ERF064 protein and pHIS2-F1R grew well in the SD/-Leu-Trp-His medium (90 mM 3-AT), indicating that CaAP2/ERF064 protein could bind to this segment (F1R) of the *CaBPR1* gene (Figure 4B).

For further confirmation, the Pro_CaBPR1_:CaBPR1 transgenic tobacco plants (Figure S3) were subjected to infiltration with expression constructs of CaAP2/ERF064 and its deletion mutants. As shown in Figure 4C, the expression level of *CaBPR1* under the treatment of *CaAP2/ERF064* was 27.57-fold compared to control at three days post-infiltration, while the expression level of *CaBPR1* under the treatment of N2, C1, C2, and C3 mutants were 6.61, 14.66, 17.50, and 5.51-fold, respectively. Both *CaAP2/ERF064* and its C2 mutant significantly induced the transcription level of *CaBPR1* in Pro_CaBPR1_:CaBPR1 transgenic tobacco plants. These results demonstrated that CaAP2/ERF064 protein can activate the expression of the *CaBPR1* gene in plants.

### 3.4. Silencing of CaAP2/ERF064 in Pepper Enhances the Susceptibility to P. capsici

**Figure 5:**
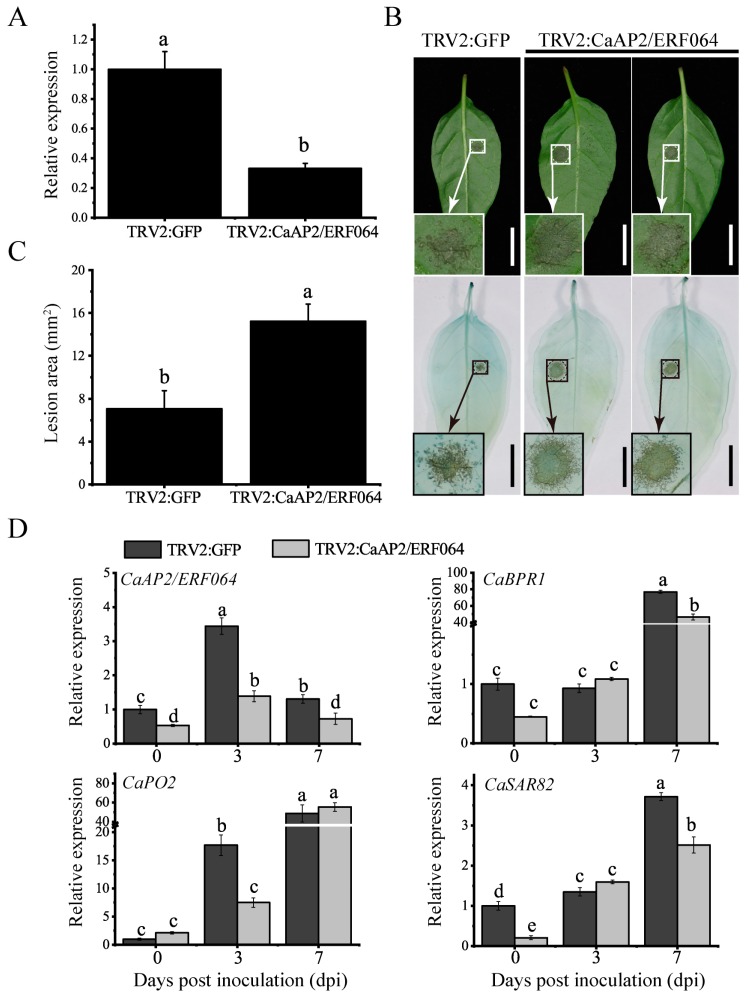
Virus-induced gene silencing (VIGS) of *CaAP2/ERF064* in pepper enhanced susceptibility to *P. capsici* infection. (**A**) Silencing efficiency of *CaAP2/ERF064* in silenced and control plants. (**B**) Disease symptom on pepper leaves at 4 dpi. (**C**) Analysis of leave lesion and quantified using ImageJ software. (**D**) Expression analysis of defense-related genes in *CaAP2/ERF064*-silenced and control plants. Values are means ± SD. Small letters indicate a significant difference (LSD, *p* < 0.05).

To evaluate a potential function for *CaAP2/ERF064* in resistance to *P. capsici*, the VIGS technique was used to silence *CaAP2/ERF064* in the pepper cultivar Y5, which is highly resistance to *P. capsici*. To visually verify the success of *CaAP2/ERF064*-silencing, a TRV2:CaPDS vector (positive control) was used for the knock-down of the *CaPDS* gene, which produced a typical white color in the leaves, as a mark of photo-bleaching phenotype. Additionally, the TRV2:GFP vector was used as a negative control. After six weeks of infiltration, the *CaPDS*-silenced plants showed photo-bleaching phenotypes in the leaves, demonstrating the success of the VIGS (Figure S4). At the same time, the silencing efficiency of *CaAP2/ERF064* was examined using qRT-PCR analysis, which revealed that the *CaAP2/ERF064* gene in TRV2:CaAP2/ERF064 (*CaAP2/ERF064*-silenced) plants was 67% lower than TRV2:GFP (negative control) plants (Figure 5A). To evaluate the resistance of *CaAP2/ERF064*-silenced Y5 plants to *Phytophthora* blight disease, the detached leaf assay was used. After four days of inoculation, the *Phytophthora* blight lesions were found on the leaves of both *CaAP2/ERF064*-silenced and control plants, but the infected area of the silenced plants was significantly larger than the control plants (Figure 5B,C). Phenotypically, trypan-blue staining showed more extensive cell death in the detached leaves of *CaAP2/ERF064*-silenced plant (Figure 5B).

Furthermore, the qRT-PCR analysis was used to investigate whether the silencing of *CaAP2/ERF064* regulated the defense-related genes (*CaBPR1* [32], *CaPO2* [33], and *CaSAR82* [34]) in pepper plants. So, a substantial decrease was observed in the expression level of the *CaAP2/ERF064* gene in the silenced plant compared to the control after *P. capsici* infection (Figure 5D). However, it was found that the expression level of *CaBPR1* in *CaAP2/ERF064*-silenced plants was significantly lower (46.39-fold) than the control (76.85-fold) at seven days post-inoculation (dpi) (Figure 5D). Moreover, the expression level of *CaSAR82* in *CaAP2/ERF064*-silenced plants was also lower (2.51-fold) than that in the control (3.71-fold) at 7 dpi, while the *CaPO2* gene only exhibited a lower (7.50-fold) expression level in *CaAP2/ERF064*-silenced plants when compared with that in the control (17.70-fold) at 3 dpi. These finding indicated that silencing of *CaAP2/ERF064* gene might alter the expression of defense-related genes and enhance the plants more prone to *P. capsici* infection.

### 3.5. Ectopic Expression of CaAP2/ERF064 Enhances Tobacco Resistance to P. capsici

**Figure 6:**
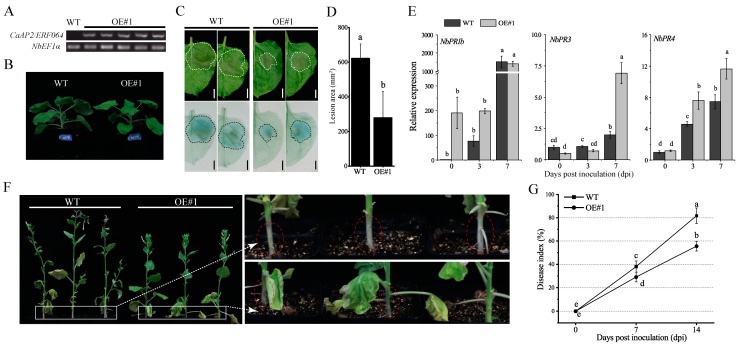
*CaAP2/ERF064-OE* tobacco plants show enhanced resistance to *P. capsici*. (**A**) Expression of *CaAP2/ERF064* in OE and wild-type (WT) plants. (**B**) Phenotype of OE and WT plants. (**C, D**) Disease symptoms and analysis of lesion area at 3 dpi. (**E**) Expression analysis of defense-related genes after *P. capsici* infection. (**F**) Phenotypes of OE and WT plants after *P. capsici* inoculation at 7 dpi. Circles indicate the shrinkage of the stem. (**G**) Disease index percentage. Values are means ± SD. Small letters indicate a significant difference (LSD, *p* < 0.05).

To further assess the potential role of *CaAP2/ERF064* in plant defense response to *P. capsici* infection, the pCaMV35S:CaAP2/ERF064-transgenic tobacco plants were generated and used for the subsequent experiment. As shown in Figure 6A,B, the *CaAP2/ERF064-OE* plants exhibited a higher expression level of *CaAP2/ERF064*, but the plant height is slightly lower than the wild-type (WT) plants. Furthermore, the results of the detached leaf assay showed that the disease lesions on the leaves of *CaAP2/ERF064-OE* plants were significantly smaller (55.12%) than the control after the infection of *P. capsici* (Figure 6C,D), and the cell death was also less extensive in the *CaAP2/ERF064-OE* plants (Figure 6C).

To further confirm the role of *CaAP2/ERF064* in disease resistance and to elucidate its possible molecular mode of action, the transcriptional responses of defense-related genes in *CaAP2/ERF064-OE* and WT plants were investigated by qRT-PCR. The defense marker gene *NbPR1b* showed a higher expression level in *CaAP2/ERF064-OE* plants as compared to WT, which is 198.23 and 76.17-fold, respectively, at 3 dpi (Figure 6E). Moreover, the chitinase gene *NbPR3* showed higher (6.92-fold) induction in *CaAP2/ERF064-OE* plants when compared with control (1.98-fold) at 7 dpi. The expression level of *NbPR4* in *CaAP2/ERF064-OE* plants was 7.61 and 11.63-fold, which was also higher than the control (4.60 and 7.44-fold) at 3 and 7 dpi, respectively. Furthermore, severe disease symptoms (wilting, yellowing of leaves, and stem shrinkage) were observed in WT plants at 7 dpi, whereas *CaAP2/ERF064-OE* plants were shorter but less affected (Figure 6F). As is evident by a calculated disease index, *CaAP2/ERF064-OE* plants showed lower infection than the control at both 7 and 14 dpi (Figure 6G). These results demonstrated that over-expression of *CaAP2/ERF064* in tobacco plants might directly participate in the defense system, at least partly through promoting expression of *PR* genes.

## 4. Discussion

Phytohormones (SA, JA, and ET) are essential signaling molecules and play a crucial role in the regulation of plant immune response to pathogens [35,36,37]. ERF genes are the downstream components of the ET signaling pathway and are recognized to integrate various phytohormone signaling pathways in plants [3]. The cross-talk between ET and other phytohormones signaling pathways resulted in a differentiated disease resistance response in plants [35]. Previously, we found that *CaAP2/ERF064* could respond to the treatment of MeJA (2.64-fold at 3 hpt) [21]. In this study, we found that the expression level of *CaAP2/ERF064* in treatment of ET + MeJA (32.29-fold) was significantly higher than that in treatment of ET (19.65-fold) and MeJA (2.64-fold) at 3 hpt. These results indicated that the transcription of *CaAP2/ERF064* is synergistically regulated by MeJA and ET signaling pathway. This is consistent with a previous study of *Arabidopsis ERF1*, which reported that this gene could be induced by ethylene and JA, as well as by both phytohormones synergistically [38].

Previous studies suggested that some ERF genes which contained the EAR domain could induce cell death when transient over-expressed in tobacco plant. For example, the *NbCD1* gene induced hypersensitive cell death when transient over-expressed in *N. benthamiana*, while the NbCD1 protein contained the EAR domain and its EAR domain in C-terminal was indispensable for triggering cell death [39]. Likewise, *NtERF3* could also cause hypersensitive cell death when over-expressed in *Nicotiana tabacum* (*N. tabacum)*. Loss of the EAR domain in NtERF3 protein did not influence its localization to the nucleus but did abrogate the cell death response [40]. Recently, *AtERF8* was found to trigger cell death when co-overexpressed with CaMV35S:HC-Pro (gene silencing suppressor) in *N. benthamiana*, and its intact EAR domain was necessary for this activity [13]. In contrast with previous studies, we found that CaAP2/ERF064 caused cell death when expressed in *N. benthamiana*, even though the protein does not contain an obvious EAR domain. The homologs of CaAP2/ERF064, such as StERF1B-l, NbERF1B-l, and SlTSRF1 proteins, could also induce cell death in *N. benthamiana*. In other studies, it was found that the homologs of *NtERF3* in rice and tobacco could trigger cell death response when over-expressed in *N. tabacum* [40,41]. However, the other *CaAP2/ERF* genes (*CaAP2/ERF049* and *CaAP2/ERF109*) could not induce the cell death in *N. benthamiana*. All of these results indicated the distinctive role of *CaAP2/ERF064* gene in cell death response.

Additionally, the CaAP2/ERF064 protein and its N2 mutant, as well as its homologs (StERF1B-l, NbERF1B-l, and SlTSRF1), proteins were located in the nucleus. Furthermore, *CaAP2/ERF064* and its homology genes belong to ERF transcription factors family and function in the nucleus. These results speculated on whether the nucleus location of CaAP2/ERF064 proteins was the inducement of cell death response. We attempted to explore this further by adding a nuclear export-signal (NES) [42] to the C-terminal of CaAP2/ERF064-GFP fusion protein but found that the fusion protein was still localized in the nucleus (Figure S5). Disruption of the nuclear localization signals (NLS) in CaAP2/ERF064 may be an effective, alternative way to approach this, while the RPW8.2 protein confers broad-spectrum resistance against powdery mildew, and the cytoplasmic localization of RPW8.2 could trigger cell death. In contrast, the RPW8.2 protein localization in the nucleus could result in plant resistance to powdery mildew [43,44].

Current research suggests that that CaAP2/ERF064 protein had the transcriptional activity and its active domain (AD) may be located in C-terminal. A previous study of the *CaAIEF1* gene also demonstrated that an AD domain is found in its C-terminal [18]. In contrast, the AD domain of the SlTSRF1 protein is located in the N-terminal [45]. In this study, we found that the CaAP2/ERF064 protein could bind to the promoter fragment (F1R) of the *CaBPR1* gene and that the transient over-expression of CaAP2/ERF064 or its deletion mutant C2 significantly increased the transcription level of *CaBPR1* in Pro_CaBPR1_:CaBPR1 transgenic tobacco plants, while the conserved AP2 domain of ERF proteins has the ability of DNA-binding [5,46]. Therefore, the AD domain of CaAP2/ERF064 protein may be located in the C-terminal, as our research showed that the C3 mutant, which contains the AD domain, exhibited strong transcriptional activity in yeast. Moreover, the C2 mutant containing both the AP2 (DNA-binding domain) and AD domains binds to the promoter and activates expression of the *CaBPR1* gene in Pro_CaBPR1_:CaBPR1 transgenic plants. These results suggested that *CaAP2/ERF064* is functioning as a transcriptional activator and could promote the expression of the *CaBPR1* gene in plants.

The loss of function or silencing of *CaAP2/ERF064* also reduced the expression of defense-related genes *CaBPR1*, *CaPO2*, and *CaSAR82* and increased the susceptibility of pepper plants to *P. capsici*. Similarly, silencing of the *CaWRKY22* gene altered the transcription level of *CaPO2* and *CaBPR1* in plants after inoculation with *Ralstonia solanacearum* [47]. Previous studies found that over-expression of *CaBPR1* enhanced resistance to *Phytophthora nicotianae*, *Ralstonia solanacearum*, and *Pseudomonas syringae* pv. *tabaci* in *N. tabacum* [48]. The decrease in the expression level of *CaBPR1* may be due to the knock-down of *CaAP2/ERF064* in TRV2:CaAP2/ERF064 plants, while *CaAP2/ERF064* is likely only one of several or many transcription factors that regulate *CaBPR1*; further research is needed to elucidate the exact role of *CaAP2/ERF064* in transcription regulation of *CaBPR1*, as well as in plant defense response to *P. capsici*.

The over-expression of *the CaAP2/ERF064* gene in tobacco plants increased the transcription of *NbPR1b*, *NbPR3*, and *NbPR4* that enhanced resistance to *P. capsici*. Additionally, the over-expression of *CaWRKY40* in *N. tabacum* also enhanced the plant defense response to *R. solanacearum* infection by upregulating the expression of *NtPR1b* and *NtPR3* [49]. *SpWRKY6* transgenic tomato plants showed enhanced resistance to *Phytophthora infestans* infection and increased the expression of *PR1*, *PR2*, *PR3*, and *PR5* [50]. However, the over-expression of *GmERF5* in soybean significantly enhanced plant resistance to *P. sojae* by increasing the expression level of *PR* (*PR10, PR1-1,* and *PR10-1*) genes [13]. Moreover, the transcript level of *PDF1.2* in *ERF96*-*RNAi Arabidopsis* plants was lower than WT, which suggests that *ERF96* plays a positive role in plant resistance to *Botrytis cinerea* [6], while *Arabidopsis* plants expressing *SlERF84* exhibited comprised immunity resistance against *Pseudomonas syringae* pv. *tomato* DC3000, and this was associated with diminished expression of *AtPR1* and *AtPR3* [51]. Recently, it was found that the over-expression of *ERF19* could repress plant pattern-triggered immunity (PTI) response and increase plant susceptibility to *Botrytis cinerea* and *Pseudomonas syringae* [52]. Furthermore, *CaAP2/ERF064*-*OE* tobacco plants are slightly shorter than WT plants, similar to the stunted phenotype reported for *Arabidopsis* plants overexpressing *AtERF14*. Similar to our observations, those *Arabidopsis* plants also showed enhanced expression of the defense-related genes *PR1*, *ChiB*, and *PDF1.2* [53]. Inappropriate activation of immune response may reduce growth of the plant [54,55]. We found that the expression level of *NbPR1b* in *CaAP2/ERF064-OE* plants was much higher than the control before inoculation (Figure 6E). Taken together, all these results indicate a vital role for *CaAP2/ERF064* in the defense mechanism against *P. capsici*.

In conclusion, the transient over-expression of *CaAP2/ERF064* with *P19* induced cell death in *N. benthamiana* leaves. Additionally, the transcription of *CaAP2/ERF064* is synergistically regulated by the MeJA and ET signaling pathway. *CaAP2/ERF064* works as a transcription activator and promotes the expression of the *CaBPR1* gene in plants. Moreover, it also positively regulates plant defense responses against *P. capsici* by modulating the transcription of *PR* genes. To elucidate the mechanisms associated with this activity, further studies are needed to identify additional target genes of *CaAP2/ERF064* and factors that participate in its specific role in cell death.

## Supplementary Materials

The following are available online at https://www.mdpi.com/2073-4425/10/7/541/s1, Table S1: Primers used in this study, Figure S1: Localization of deletion mutants of CaAP2/ERF064 protein in *N. benthamiana* epidermal cells. Scale bar represent 100 μm, Figure S2: Transient over-expression of CaAP2/ERF049 and CaAP2/ERF109 in *N. benthamiana*. EV was short for empty vector. The photograph was taken at 7 days post infiltration. Scale bar represent 1 cm, Figure S3: Detection of Pro_CaBPR1_:CaBPR1 transgenic tobacco plants. (A) Expression analysis of *CaBPR1* in Pro_CaBPR1_:CaBPR1 transgenic tobacco plants. (B) Phenotypes of WT and Pro_CaBPR1_:CaBPR1 transgenic plants, Figure S4: Phenotype of *CaPDS*-silenced pepper plants at 6 weeks post infiltration, Figure S5: Localization of CaAP2/ERF064 proteins in *N. benthamiana* epidermal cells. (A) and (B) Localization of CaAP2/ERF064-GFP-NLS and CaAP2/ERF064-GFP-NES proteins in tobacco. The Scale bar represents 100 μm.

## References

[1] Peng Y., Van Wersch R., Zhang Y. (2018). Convergent and Divergent Signaling in PAMP-Triggered Immunity and Effector-Triggered Immunity. Mol. Plant-Microbe Interact..

[2] Chisholm S.T., Coaker G., Day B., Staskawicz B.J. (2006). Host-Microbe Interactions: Shaping the Evolution of the Plant Immune Response. Cell.

[3] Huang P.Y., Catinot J., Zimmerli L. (2016). Ethylene response factors in *Arabidopsis* immunity. J. Exp. Bot..

[4] Müller M., Munné-Bosch S. (2015). Ethylene Response Factors: A Key Regulatory Hub in Hormone and Stress Signaling1. Plant Physiol..

[5] Ohme-Takagi M., Shinshi H. (1995). Ethylene-inducible DNA binding proteins that interact with an ethylene-responsive element. Plant Cell.

[6] Catinot J., Huang J.-B., Huang P.-Y., Tseng M.-Y., Chen Y.-L., Gu S.-Y., Lo W.-S., Wang L.-C., Chen Y.-R., Zimmerli L. (2015). ETHYLENE RESPONSE FACTOR 96 positively regulates *Arabidopsis* resistance to necrotrophic pathogens by direct binding to GCC elements of jasmonate- and ethylene-responsive defence genes. Plant Cell Environ..

[7] Zhao Y., Wei T., Yin K.-Q., Chen Z., Gu H., Qu L.-J., Qin G. (2012). *Arabidopsis* RAP2.2 plays an important role in plant resistance to *Botrytis cinerea* and ethylene responses. New Phytol..

[8] Son G.H., Wan J., Kim H.J., Nguyen X.C., Chung W.S., Hong J.C., Stacey G. (2012). Ethylene-Responsive Element-Binding Factor 5, ERF5, Is Involved in Chitin-Induced Innate Immunity Response. Mol. Plant-Microbe Interact..

[9] McGrath K.C., Dombrecht B., Manners J.M., Schenk P.M., Edgar C.I., MacLean D.J., Scheible W.-R., Udvardi M.K., Kazan K. (2005). Repressor-and Activator-Type Ethylene Response Factors Functioning in Jasmonate Signaling and Disease Resistance Identified via a Genome-Wide Screen of *Arabidopsis* Transcription Factor Gene Expression. Plant Physiol..

[10] Ohta M., Matsui K., Hiratsu K., Shinshi H., Ohme-Takagi M. (2001). Repression Domains of Class II ERF Transcriptional Repressors Share an Essential Motif for Active Repression. Plant Cell.

[11] Tian Z., He Q., Wang H., Liu Y., Zhang Y., Shao F., Xie C. (2015). The Potato ERF Transcription Factor StERF3 Negatively Regulates Resistance to *Phytophthora infestans* and Salt Tolerance in Potato. Plant Cell Physiol..

[12] Dong L.D., Cheng Y.X., Wu J.J., Cheng Q., Li W.B., Fan S.J., Jiang L.Y., Xu Z.L., Kong F.J., Zhang D.Y. (2015). Overexpression of GmERF5, a new member of the soybean EAR motif-containing ERF transcription factor, enhances resistance to *Phytophthora sojae* in soybean. J. Exp. Bot..

[13] Cao F.Y., DeFalco T.A., Moeder W., Li B., Gong Y., Liu X.-M., Taniguchi M., Lumba S., Toh S., Shan L. (2018). *Arabidopsis* ETHYLENE RESPONSE FACTOR 8 (ERF8) has dual functions in ABA signaling and immunity. BMC Plant Boil..

[14] Cao F.Y., Khan M., Taniguchi M., Mirmiran A., Moeder W., Lumba S., Yoshioka K., Desveaux D. (2019). A host–pathogen interactome uncovers phytopathogenic strategies to manipulate plant ABA responses. Plant J..

[15] Lee J.-H., Hong J.-P., Oh S.-K., Lee S., Choi D., Kim W. (2004). The ethylene-responsive factor like protein 1 (CaERFLP1) of hot pepper (*Capsicum annuum* L.) interacts in vitro with both GCC and DRE/CRT sequences with different binding affinities: Possible biological roles of CaERFLP1 in response to pathogen infection and high salinity conditions in transgenic tobacco plants. Plant Mol. Boil..

[16] Yi S.Y., Kim J.-H., Joung Y.-H., Lee S., Kim W.-T., Yu S.H., Choi D. (2004). The Pepper Transcription Factor CaPF1 Confers Pathogen and Freezing Tolerance in *Arabidopsis*. Plant Physiol..

[17] Jin J.H., Zhang H.X., Tan J.Y., Yan M.J., Li D.W., Khan A., Gong Z.H. (2016). A New Ethylene-Responsive Factor CaPTI1 Gene of Pepper (*Capsicum annuum* L.) Involved in the Regulation of Defense Response to *Phytophthora capsici*. Front. Plant Sci..

[18] Hong E., Lim C.W., Han S.-W., Lee S.C. (2017). Functional Analysis of the Pepper Ethylene-Responsive Transcription Factor, CaAIEF1, in Enhanced ABA Sensitivity and Drought Tolerance. Front. Plant Sci..

[19] Kamoun S., Furzer O., Jones J.D., Judelson H.S., Ali G.S., Dalio R.J., Roy S.G., Schena L., Zambounis A., Panabieres F. (2015). The Top 10 oomycete pathogens in molecular plant pathology. Mol. Plant Pathol..

[20] Lamour K.H., Stam R., Jupe J., Huitema E. (2012). The oomycete broad-host-range pathogen *Phytophthora capsici*. Mol. Plant Pathol..

[21] Jin J.-H., Wang M., Zhang H.-X., Khan A., Wei A.-M., Luo D.-X., Gong Z.-H. (2018). Genome-wide identification of the AP2/ERF transcription factor family in pepper (*Capsicum annuum* L.). Genome.

[22] Wang Y., Meng Y., Zhang M., Tong X., Wang Q., Sun Y., Quan J., Govers F., Shan W. (2011). Infection of *Arabidopsis thaliana* by *Phytophthora parasitica* and identification of variation in host specificity. Mol. Plant Pathol..

[23] Du Y., Mpina M.H., Birch P.R.J., Bouwmeester K., Govers F. (2015). Phytophthora infestans RXLR Effector AVR1 Interacts with Exocyst Component Sec5 to Manipulate Plant Immunity. Plant Physiol..

[24] Zhang H.X., Ali M., Feng X.H., Jin J.H., Huang L.J., Khan A., Lv J.G., Gao S.Y., Luo D.X., Gong Z.H. (2018). A Novel Transcription Factor CaSBP12 Gene Negatively Regulates the Defense Response against *Phytophthora capsici* in Pepper (*Capsicum annuum* L.). Int. J. Mol. Sci..

[25] Wang J.-E., Liu K.-K., Li D.-W., Zhang Y.-L., Zhao Q., He Y.-M., Gong Z.-H. (2013). A Novel Peroxidase CanPOD Gene of Pepper Is Involved in Defense Responses to *Phytophtora capsici* Infection as well as Abiotic Stress Tolerance. Int. J. Mol. Sci..

[26] Mendel R.R., Schiemann J., Simoens C. (1987). High meiotic stability of a foreign gene introduced into tobacco by *Agrobacterium*-mediated transformation. Mol. Genet. Genom..

[27] Choi D.S., Hwang I.S., Hwang B.K. (2012). Requirement of the Cytosolic Interaction between PATHOGENESIS-RELATED PROTEIN10 and LEUCINE-RICH REPEAT PROTEIN1 for Cell Death and Defense Signaling in Pepper. Plant Cell.

[28] Livak K.J., Schmittgen T.D. (2001). Analysis of relative gene expression data using real-time quantitative PCR and the 2(T)(-Delta Delta C) method. Methods.

[29] Scholthof H.B. (2006). The Tombusvirus-encoded P19: From irrelevance to elegance. Nat. Rev. Genet..

[30] Silhavy D., Burgyán J. (2004). Effects and side-effects of viral RNA silencing suppressors on short RNAs. Trends Plant Sci..

[31] Hong J.K., Lee S.C., Hwang B.K. (2005). Activation of pepper basic PR-1 gene promoter during defense signaling to pathogen, abiotic and environmental stresses. Gene.

[32] Kim Y.J., Hwang B.K. (2000). Pepper gene encoding a basic pathogenesis-related 1 protein is pathogen and ethylene inducible. Physiol. Plant..

[33] Choi H.W., Kim Y.J., Lee S.C., Hong J.K., Hwang B.K. (2007). Hydrogen Peroxide Generation by the Pepper Extracellular Peroxidase CaPO2 Activates Local and Systemic Cell Death and Defense Response to Bacterial Pathogens. Plant Physiol..

[34] Lee S.C., Hwang B.K. (2003). Identification of the pepper SAR8.2 gene as a molecular marker for pathogen infection, abiotic elicitors and environmental stresses in *Capsicum annuum*. Planta.

[35] Broekaert W.F., Delauré S.L., De Bolle M.F., Cammue B.P. (2006). The Role of Ethylene in Host-Pathogen Interactions. Annu. Rev. Phytopathol..

[36] Pieterse C.M., Van Der Does D., Zamioudis C., Leon-Reyes A., Van Wees S.C. (2012). Hormonal Modulation of Plant Immunity. Annu. Rev. Cell Dev. Boil..

[37] Kunkel B.N., Brooks D.M. (2002). Cross talk between signaling pathways in pathogen defense. Curr. Opin. Plant Boil..

[38] Lorenzo O., Piqueras R., Sánchez-Serrano J.J., Solano R. (2003). ETHYLENE RESPONSE FACTOR1 integrates signals from ethylene and jasmonate pathways in plant defense. Plant Cell.

[39] Bin Nasir K.H., Takahashi Y., Ito A., Saitoh H., Matsumura H., Kanzaki H., Shimizu T., Ito M., Fujisawa S., Sharma P.C. (2005). High-throughput in planta expression screening identifies a class II ethylene-responsive element binding factor-like protein that regulates plant cell death and non-host resistance. Plant J..

[40] Ogata T., Kida Y., Arai T., Kishi Y., Manago Y., Murai M., Matsushita Y. (2012). Overexpression of tobacco ethylene response factor NtERF3 gene and its homologues from tobacco and rice induces hypersensitive response-like cell death in tobacco. J. Gen. Plant Pathol..

[41] Ogata T., Kida Y., Tochigi M., Matsushita Y. (2013). Analysis of the cell death-inducing ability of the ethylene response factors in group VIII of the AP2/ERF family. Plant Sci..

[42] Matsushita T., Mochizuki N., Nagatani A. (2003). Dimers of the N-terminal domain of phytochrome B are functional in the nucleus. Nature.

[43] Huang Y.Y., Shi Y., Lei Y., Li Y., Fan J., Xu Y.J., Ma X.F., Zhao J.Q., Xiao S.Y., Wang W.M. (2014). Functional identification of multiple nucleocytoplasmic trafficking signals in the broad-spectrum resistance protein RPW8.2. Planta.

[44] Huang Y.Y., Zhang L.L., Ma X.F., Zhao Z.X., Zhao J.H., Zhao J.Q., Fan J., Li Y., He P., Xiao S. (2018). Multiple intramolecular trafficking signals in RESISTANCE TO POWDERY MILDEW 8.2 are engaged in activation of cell death and defense. Plant J..

[45] Zhang H., Li W., Chen J., Yang Y., Zhang Z., Zhang H., Wang X.C., Huang R. (2007). Transcriptional activator TSRF1 reversely regulates pathogen resistance and osmotic stress tolerance in tobacco. Plant Mol. Biol..

[46] Licausi F., Perata P., Ohme-Takagi M., Ohme-Takagi M. (2013). APETALA2/Ethylene Responsive Factor (AP2/ERF) transcription factors: Mediators of stress responses and developmental programs. New Phytol..

[47] Hussain A., Li X., Weng Y., Liu Z., Ashraf M.F., Noman A., Yang S., Ifnan M., Qiu S., Yang Y. (2018). CaWRKY22 Acts as a Positive Regulator in Pepper Response to *Ralstonia Solanacearum* by Constituting Networks with CaWRKY6, CaWRKY27, CaWRKY40, and CaWRKY58. Int. J. Mol. Sci..

[48] Sarowar S., Kim Y.J., Kim E.N., Kim K.D., Hwang B.K., Islam R., Shin J.S. (2005). Overexpression of a pepper basic pathogenesis-related protein 1 gene in tobacco plants enhances resistance to heavy metal and pathogen stresses. Plant Cell Rep..

[49] Dang F.F., Wang Y.N., Yu L., Eulgem T., Lai Y., Liu Z.Q., Wang X., Qiu A.L., Zhang T.X., Lin J. (2013). CaWRKY40, a WRKY protein of pepper, plays an important role in the regulation of tolerance to heat stress and resistance to *Ralstonia solanacearum* infection. Plant Cell Environ..

[50] Hong Y., Cui J., Liu Z., Luan Y. (2018). SpWRKY6 acts as a positive regulator during tomato resistance to *Phytophthora infestans* infection. Biochem. Biophys. Res. Commun..

[51] Li Z., Tian Y., Xu J., Fu X., Gao J., Wang B., Han H., Wang L., Peng R., Yao Q. (2018). A tomato ERF transcription factor, SlERF84, confers enhanced tolerance to drought and salt stress but negatively regulates immunity against *Pseudomonas syringae* pv. tomato DC3000. Plant Physiol. Biochem. PPB.

[52] Huang P.Y., Zhang J., Jiang B., Chan C., Yu J.H., Lu Y.P., Chung K., Zimmerli L. (2019). NINJA-associated ERF19 negatively regulates *Arabidopsis* pattern-triggered immunity. J. Exp. Bot..

[53] Onate-Sanchez L., Anderson J.P., Young J., Singh K.B. (2007). AtERF14, a member of the ERF family of transcription factors, plays a nonredundant role in plant defense. Plant Physiol..

[54] Huot B., Yao J., Montgomery B.L., He S.Y. (2014). Growth–Defense Tradeoffs in Plants: A Balancing Act to Optimize Fitness. Mol. Plant.

[55] Ning Y., Liu W., Wang G.-L. (2017). Balancing Immunity and Yield in Crop Plants. Trends Plant Sci..

