# Supplementary material for: The CaAP2/ERF064 Regulates Dual Functions in Pepper: Plant Cell Death and Resistance to Phytophthora capsici
Source: Genes (Basel). 2019 Jul 17;10(7):541. doi: 10.3390/genes10070541 (PMC6678779; doi:10.3390/genes10070541)
Supplement: Supplementary file 1 [file genes-10-00541-s001.pdf]

genes

MDPI

Table S1. Primers used in this study.

Gene	Primer name	Sequence (5′-3′)	Genbank No.	Purposes
CaAP2/ERF064	CDS-F	GGGGTACCATGGACTCAACTCCTTCTTC	XM_016712649.1	Transient expression
	CDS-R	GCTCTAGACCATGGACTAAAATAAGTAG		
	C1-F	GGGGTACCATGTCGTACCGAGGTGTTAG		
	C3-F	GGGGTACCATGGAGACAGTTAGAGAGT		
	N2-R	GCTCTAGACACTGGGAAATTCAGTA		
	N1-R	GCTCTAGACTTCTTCTTACCGGGT		
	AD-F	CGGGATCCATATGGACTCAACTCCTTC		Yeast one hybrid
	AD-R	CCGCTCGAGTTACCATGGACTAAAATA		
	BD-CDS-F	TCCCCCGGGGATGGACTCAACTCCTT		Transcriptional activity analysis
	BD-C1-F	TCCCCCGGGGTCGTACCGAGGTGTTAG		
	BD-C3-F	TCCCCCGGGGGAGACAGTTAGAGAGT		
	BD-N1-R	AACTGCAGTTACTTCTTCTTACCGGGT		
	BD-N2-R	AACTGCAGTTACACTGGGAAATTCAGTA		
	BD-CDS-R	AACTGCAGTTACCATGGACTAAAATAAG		
	OE-F	GCTCTAGAATGGACTCAACTCCTTCTTC		Over-expression
	OE-R	GGGGTACCTTACCATGGACTAAAATAAGTA		
	qRT-F	CAACTCCTTCTTCTTGCTCTTC		qRT-PCR
	qRT-R	CGCCTCCTAACACCTCGGTA		
	VIGS-F	GCTCTAGACACATGGACCAAGACTGTTCTC		VIGS
	VIGS-R	GGGGTACCTTACCATGGACTAAAATAAGTA		
CaAP2/ERF049	CDS-F	GGGGTACCATGGTTCCAACTCACCAAAGT	KJ690096.1	Transient expression
	CDS-R	GCTCTAGAGAGCGCCAAGAAATTCTC		
CaAP2/ERF109	CDS-F	GGGGTACCATGTCGGGCAACTCATCAAG	XM_016717675.1	Transient expression
	CDS-R	GCTCTAGATGATGGAGTCATTAGAAGCT		
StERF1B-l	CDS-F	GGGGTACCATGGATTCTTCTTCTTGTTC	XM_006354438.2	Transient expression
	CDS-R	GCTCTAGACCATGGACTAAAATAAGTTG		
SlTSRF1	CDS-F	GGGGTACCATGGATTCTTCTTCTTCTTC	AAN32899.1	Transient expression
	CDS-R	GCTCTAGACCATGGACTAAAATAAGTTG		

NbERF1B-l	CDS-F	GGGGTACCATGGATTCTTCTTCTTGTTC	Niben101scf07761g02006.1	Transient expression
	CDS-R	GCTCTAGACCATGGTAGAAAAGCTTCATTAC		
NbCD1	CDS-F	GGGGTACCATGGCGCCGAAAGAAAAAGG	AB196362.1	Transient expression
	CDS-R	GCTCTAGACATGTTTTCCGGCGGAGGAAG		
PcINF1	CDS-F	GGGGTACCATGAACTTCCGTGCTCTGTT	JX948084.1	Transient expression
	CDS-R	GCTCTAGATTACAGCGACGCGCACGTG		
CaBPR1	promoter-F	CCCAAGCTTGTGCATTATTAGAAACTTGTGGGT	AY560589.1	Tobacco transformation
	CDS-R	CGGGATCCGACATCAGTTGGAAGTTCC		
	pHIS-F1	CCGGAATTCGGGTCAATGTAGTACTCC		Yeast one hybrid
	pHIS-R	CGACGCGTCTTGAATGAATGGGTTGC		
	qRT-F	CAGGATGCAACACTCTGGTGG		qRT-PCR
	qRT-R	ATCAAAGGCCGGTTGGTC		
CaPO2	qRT-F	TGATTGCTTTGTTCAGGGTT	DQ489711.1	qRT-PCR
	qRT-R	ATGATGGACCTCCAACGAGA		
CaSAR82	qRT-F	CAGGGAGATGAATTCTGAGGC	AF313766.1	qRT-PCR
	qRT-R	CATATGAACCTCTATGGATTTCTG		
CaUBI3	qRT-F	TGTCCATCTGCTCTCTGTTG	AY486137.1	qRT-PCR
	qRT-R	CACCCCAAGCACAATAAGAC		
GUS	qRT-F	CTCATTACGGCAAAGTGTGG		qRT-PCR
	qRT-R	CATTACGCTGCGATGGATTC		
NbPR3	qRT-F	GACAACAAGCCATCTTCCCA	X51425.1	qRT-PCR
	qRT-R	ACAGTCCAAGTTTTCCCCAG		
NbPR4	qRT-F	TGATGGTGGCAATGGCGGCG	X58547.1	qRT-PCR
	qRT-R	GTTCCTGTTCCTGTGTTCGT		
NbPR1b	qRT-F	ATGGGATACTCCAAAACAT	X66942.1	qRT-PCR
	qRT-R	CACCAGCGGCGTTGAGTTGA		
NbEF1α	qRT-F	CGTGAGCGTGGTATCACCATT	AY206004.1	qRT-PCR
	qRT-R	GTGAAAGCAAGCAATGCGTG		

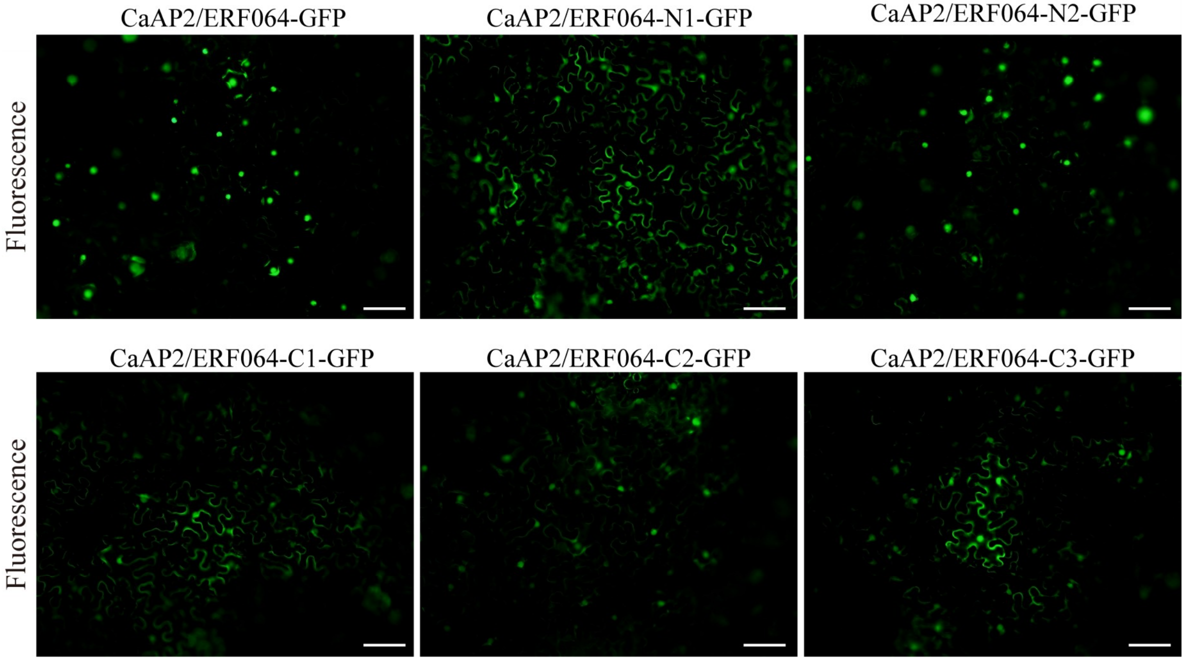

Figure S1. Localization of deletion mutants of CaAP2/ERF064 protein in *N. benthamiana* epidermal cells. Scale bar represent 100 μm.

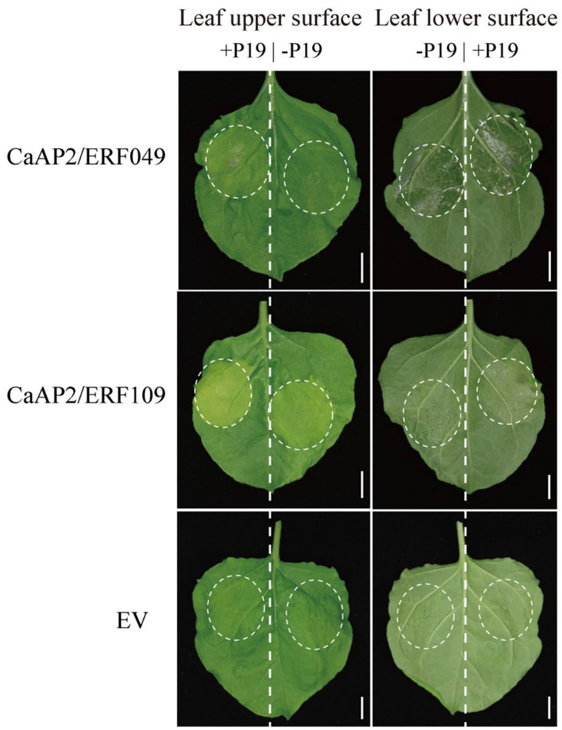

Figure S2. Transient over-expression of *CaAP2/ERF049* and *CaAP2/ERF109* in *N. benthamiana*. EV was short for empty vector. The photograph was taken at 7 days post infiltration. Scale bar represent 1 cm.

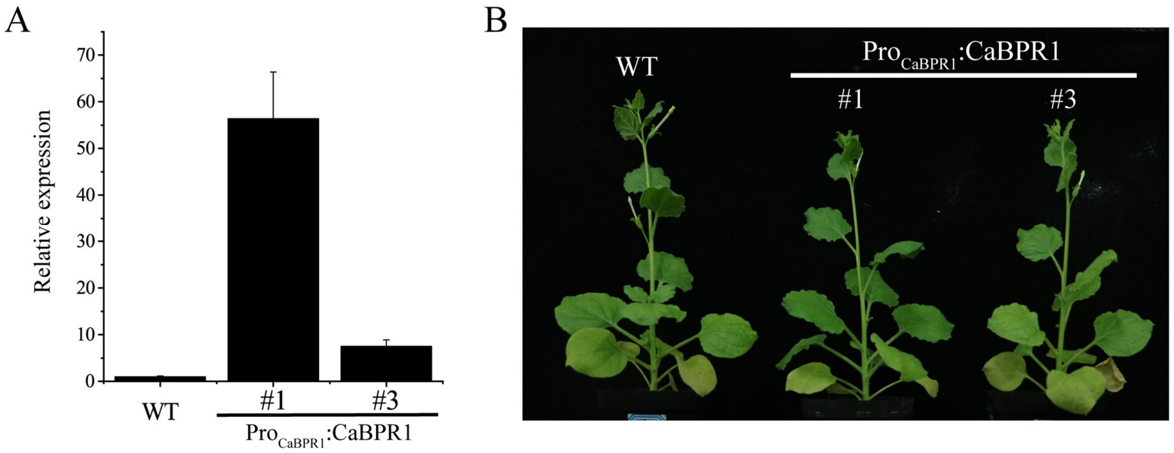

Figure S3. Detection of Pro$_{CaBPR1}$:CaBPR1 transgenic tobacco plants. (A) Expression analysis of *CaBPR1* in Pro$_{CaBPR1}$:CaBPR1 transgenic tobacco plants. (B) Phenotypes of WT and Pro$_{CaBPR1}$:CaBPR1 transgenic plants.

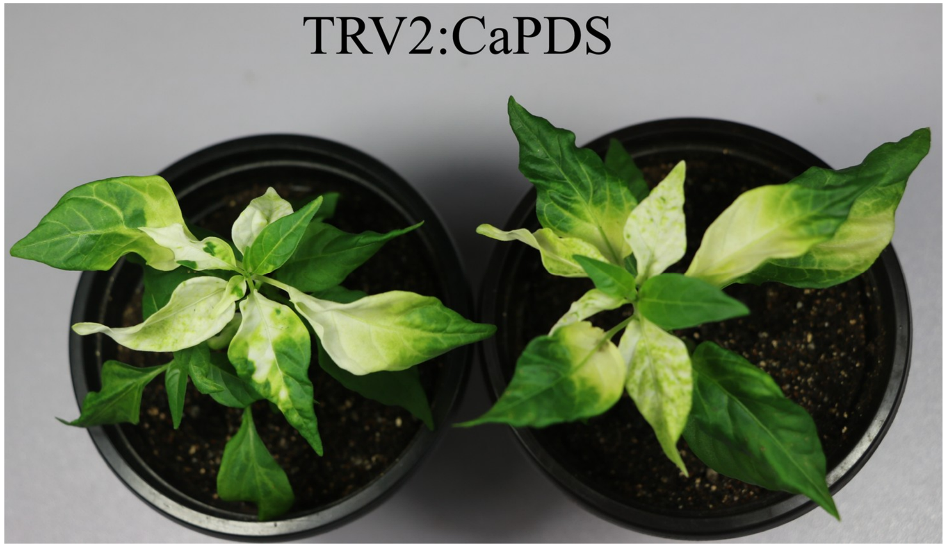

Figure S4. Phenotype of *CaPDS*-silenced pepper plants at 6 weeks post infiltration.

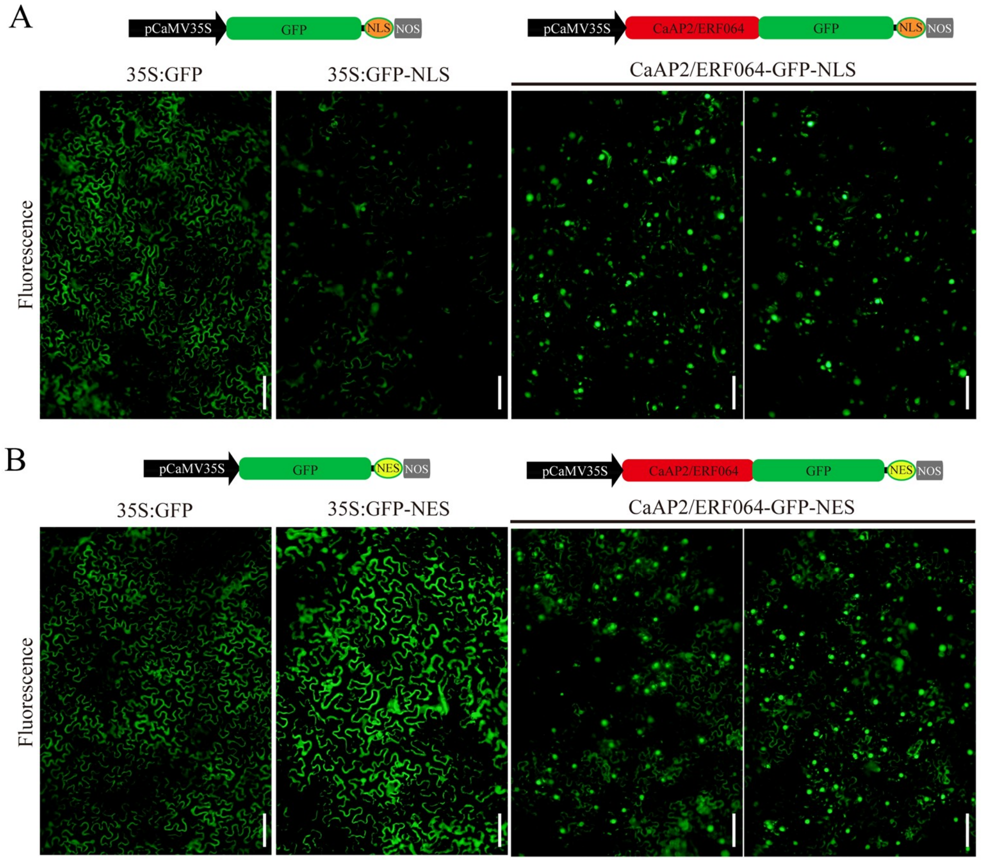

Figure S5. Localization of CaAP2/ERF064 proteins in *N. benthamiana* epidermal cells. (A) and (B) Localization of CaAP2/ERF064-GFP-NLS and CaAP2/ERF064-GFP-NES proteins in tobacco. The Scale bar represents 100 μm.
